# Proteomics of protein post-translational modifications implicated in neurodegeneration

**DOI:** 10.1186/2047-9158-3-23

**Published:** 2014-10-30

**Authors:** Ru-Jing Ren, Eric B Dammer, Gang Wang, Nicholas T Seyfried, Allan I Levey

**Affiliations:** Department of Neurology,Center for Neurodegenerative Diseases, Emory University School of Medicine, Atlanta, GA 30322 USA; Department of Biochemistry, Center for Neurodegenerative Diseases, Emory University School of Medicine, Atlanta, GA 30322 USA; Department of Pharmacology, Center for Neurodegenerative Diseases, Emory University School of Medicine, Atlanta, GA 30322 USA; Emory Proteomics Service Center, Center for Neurodegenerative Diseases, Emory University School of Medicine, Atlanta, GA 30322 USA

**Keywords:** Proteomics, Protein posttranslational modifications, Neurodegeneration, Alzheimer’s disease, Parkinson’s disease

## Abstract

Mass spectrometry (MS)-based proteomics has developed into a battery of approaches that is exceedingly adept at identifying with high mass accuracy and precision any of the following: oxidative damage to proteins (redox proteomics), phosphorylation (phosphoproteomics), ubiquitination (diglycine remnant proteomics), protein fragmentation (degradomics), and other posttranslational modifications (PTMs). Many studies have linked these PTMs to pathogenic mechanisms of neurodegeneration. To date, identifying PTMs on specific pathology-associated proteins has proven to be a valuable step in the evaluation of functional alteration of proteins and also elucidates biochemical and structural explanations for possible pathophysiological mechanisms of neurodegenerative diseases. This review provides an overview of methods applicable to the identification and quantification of PTMs on proteins and enumerates historic, recent, and potential future research endeavours in the field of proteomics furthering the understanding of PTM roles in the pathogenesis of neurodegeneration.

**Electronic supplementary material:**

The online version of this article (doi:10.1186/2047-9158-3-23) contains supplementary material, which is available to authorized users.

## 1. Introduction

Neurodegenerative diseases representing a diverse spectrum of disorders differ in molecular etiology and progression, as represented by Alzheimer’s disease (AD), Parkinson’s disease (PD), dementia with Lewy bodies (DLB), Huntington’s disease (HD), frontotemporal lobar dementia (FTLD), amyotrophic lateral sclerosis (ALS), and others. Such diseases present in patients as progressively worsening symptoms, sometimes with overlap between diagnoses such that they are ultimately and unambiguously identified only by signature molecular neuropathology found at autopsy. For example, cognitive impairment, behavioral deficits, motor sensory dysfunction and other deficits can overlap due to degeneration of specific neural circuits and underlying death and/or dysfunction of neurons, glia, and/or vasculature. The pathogenic mechanism resulting in the onset and progression of each disease is often associated with genetic variants and mutations and interactions with environmental impacts, lifestyle risk factors, and slowly evolving molecular changes due to aging [1, 2]. Despite the broad range of neurodegenerative clinical and pathological phenotypes, there are several common pathogenic mechanisms. An emerging theme for most diseases is the accumulation of misfolded peptide or protein aggregation and aggregate deposition within areas of the cerebral cortex, basal ganglia, and/or spinal cord, although the relationship between clinical phenotype and protein dysfunction have not been completely elucidated thus far [3, 4]. Protein homeostasis and folding capacity, sometimes referred to as proteostasis, has been suggested as a possible common pathway that is progressively dysregulated with aging and neurodegenerative pathogenesis [5, 6]. Protein forms which emerge post-translationally through modification of protein residues can have starkly different properties due to a single post-translational modification (PTM), for example phosphorylation of Tau in neurofibrillary tangles, or cleavage and PTM of amyloid precursor protein (APP) to yield modified amyloid beta peptides.

Mass spectrometry (MS) is an emerging platform developed to identify and quantify proteins and exact mass/charge (m/z) shifts due to PTMs, of either intact proteins, or peptides that derive from those proteins. MS-based proteomics has provided a powerful means to profile complex protein mixtures as tens to hundreds of thousands of peptides in bottom-up proteomics, and will open the door to the identification of a billion estimated proteoforms due to specific combinations of PTMs on intact proteins across different cell types [7], which can also be accessed with top-down MS approaches [8]. A number of research avenues to investigate neurodegeneration at different levels [e.g. in tissue, subcellular and biochemical fractions, and biomarkers in the extracellular milieu, including plasma or cerebrospinal fluid (CSF)] with a variety of specific proteomic methods have been involved in successful biomarker discovery, drug target development and elucidation of pathogenic mechanisms in the past 20 years [9–17]. The purpose of this review is to first present an overview of general methodologies applicable to the study of post-translational modifications (PTMs) of proteins, and second, we provide references which highlight the importance, perhaps central, of PTMs in characterizing pathogenic processes of neurodegeneration and imbalances in proteostasis.

## 2. Proteomic approaches geared toward PTM identification and quantitation

### 2.1 Biophysical and biochemical separations

**Figure 1:**
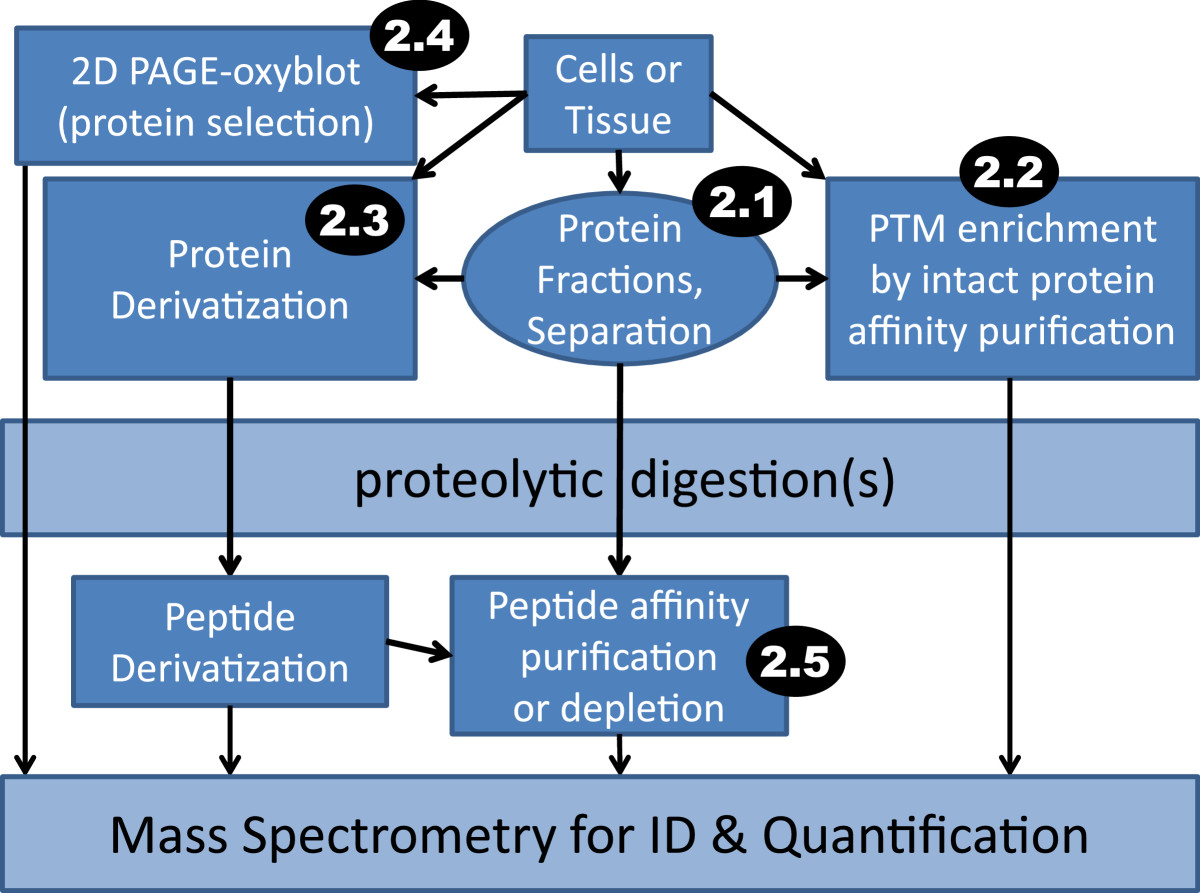
**Workflows for PTM identification (ID) and quantification by MS.** Inset black ovals with white text specify review sections pertaining to these methods.

Biophysical and biochemical separations offer a means of reducing complexity of protein samples prior to MS, and of homing into PTMs in different cell types or compartments. Subcellular fractionation has been successfully employed to look at AD-associated differences in blood derived [18, 19] or brain tissue-derived membrane proteomes [20] and at neuron-specific nuclear proteomes from post-mortem brain arrived at after fluorescence activated cell sorting (FACS) [21]. Both membrane and nuclear proteomes are accessed through ultracentrifugation, whereas other separations may involve microdissection or other means of selective isolation of affected regional samples from tissue, such as laser capture of cells with high aggregate protein burden analysed subsequently by MS [22]. Biochemical strategies are also useful for purification of detergent-resistant inclusions and/or plaques [23] which can occur not only extracellularly, but also in the nucleus, cytosol, or mitochondria under conditions ranging from physiological to pathophysiological [24–26]. Thus, fractionation of cells and tissue enables more sensitive MS detection for low abundance proteins and promises to open up avenues for specific identification of PTMs isolated to cell types and compartments (Figure 1).

### 2.2 Intact protein affinity purification

Affinity purification is an approach whereby proteins or peptides can be enriched via a binding property or other physical characteristic that allows selective interaction with specific target(s). We first discuss a couple of affinity approaches that purify intact proteins; these serve only as examples of a number of approaches similar in principle.

#### 2.2.1 Tandem ubiquitin binding entities access the ubiquitome

The ubiquitin-proteasome system is linked to many neurodegenerative diseases as it plays an essential role in clearance of many pathogenic proteins, and is discussed in detail in the context of neurodegeneration in section 3. Ubiquitination (or ubiquitylation) events are labile due to their propensity to signal for protein degradation, making detection challenging. Tandem ubiquitin binding entities (TUBEs) have been developed for the protection and enrichment of ubiquitinated proteins. TUBEs have increased affinity of up to 1000-fold for poly-ubiquitin chains over a single ubiquitin binding associated (UBA) domain, and protect ubiquitinated proteins from the disruption mediated by both proteasome and de-ubiquitinating enzymes [27, 28]. Thus, TUBEs allow for the detection of relatively low abundance ubiquitinated proteins that cannot be detected with previous technologies. Some TUBEs also have specificity for enrichment of proteins with specific topologies of ubiquitin chains attached, and it is not inconceivable that some may exist which have affinity for mixed chains of ubiquitin and small ubiquitin-like modifier (SUMO), where the latter protein and other ubiquitin-like modifiers provide an even greater challenge for unambiguous identification of attachment sites [29].

Generally, after TUBE-based enrichment of ubiquitin or ubiquitin/SUMO proteins and tryptic preprocessing of proteins into peptides, MS/MS is the ideal downstream application for identification of the attachment site remnant diglycine tag that results from cleavage of the attached C-terminus of ubiquitin to protein N-termini or, more usually, to lysine residue side chains of peptides. The diglycine tag from ubiquitin is left on both ubiquitination substrates and ubiquitin and SUMO residues themselves, in polyubiquitin or mixed ubiquitin/SUMO chains. There are eight attachment sites for polyubiquitination on ubiquitin itself (the best characterized being K48 and K63) which can be monitored and quantified by tandem MS (MS/MS) approaches [30–32], but tens of thousands of attachment sites across the proteome have recently been identified [33, 34], albeit following a peptide affinity approach which we discuss below, rather than using TUBEs, which nonetheless hold promise for this application. There are also three commercially available polyubiquitin chain-specific antibodies developed via phage display which are capable of immunoprecipitation of intact protein ubiquitin conjugates that can be used to profile biologically distinct ubiquitination substrates and/or associated ubiquitin linkages [35, 36].

#### 2.2.2 Lectin affinity chromatography for glycosylated proteins

Changes in glycosylation of proteins have been linked to neurodegeneration and reviewed in the context of MS-based methods in detail as recently as 2010, and an inventory therein confirms that glycoproteins are particularly well represented in CSF and brain [37]. Compared to traditional purification methods using gel separation of proteins, lectin affinity chromatography for glycosylated proteins is a method for the large-scale identification of N-linked glycoproteins from complex biological samples [38]. This approach yields indirect information on the glycan structure via lectin binding specificity for structurally variable glycans attached to glycoproteins; for example, concanavalin A (Con-A) and wheat germ agglutinin have specificity for mannose and N-acetylglucosamine, respectively [39]. Thus, lectin affinity chromatography represents a valuable tool for glycoproteome studies, reducing sample complexity and enriching glycoprotein content in samples prior to their identification by MS. The numbers of peptides identified by this method with multiple glycosylated sites will probably continue to increase with better instrumentation [40]. Glycans, which do not easily fragment into identifiable MS/MS fingerprints, are usually cleaved by an enzyme such as peptide N-glycanase (PNGase), which results in a 1 Da shift in the mass of asparagine residue attachment sites, which are converted by the enzyme to aspartate. This shift can be disambiguated as a glycan attachment site rather than an asparagine deamidation event by performing PNGase digestion in the presence of heavy (^18^O) labelled water. Con-A [41] and wheat germ agglutinin [42] have each been employed to look at changes in glycoproteins of the hippocampus and inferior parietal lobe in AD. It is worth noting that Con-A has a large hydrophobic domain that, under certain circumstances, can bind non-glycosylated proteins, leading to false positive identification of glycoproteins.

### 2.3 Derivatization based approaches

Derivatization can be used both to identify PTMs and for quantitative proteomics, where often all peptides are tagged chemically, usually with different stable isotope labels detectable by MS or MS/MS [43]. We focus here on the former class of approaches, but there is overlap in these methods. Quantitative labelling approaches also improve the accuracy and confidence of PTMs identified across samples processed by MS with different labels. Derivatization by multiplex isobaric tags for pooled analysis and quantitation of multiple samples in a single MS run had been plagued by compression effects affecting the quantitative accuracy until recently, when novel MS^3^-based or other approaches solved the problem [44, 45]. A perfect demonstration of the overlap between derivatization for both PTM identification and peptide-level quantitation comes from Robinson and Evans, who recently developed a novel workflow combining N-terminal stable isotope labelled dimethyl tagging with isobaric tagging on the C-terminal residue of LysC-derived peptides for global peptide sample multiplexing and quantitation [46], and adapted the approach to specifically derivatize an amine group onto 3-nitrotyrosine modified residues on amine-blocked peptides with the amine-reactive isobaric tag in their original publication on this approach [47], for which they coined the term “combined precursor isotopic labelling and isobaric tagging” (cPILOT).

#### 2.3.1 Endogenous N- or C-terminal peptide identification

Endogenous protein cleavage underlies the generation of pathological amyloid peptides from amyloid precursor protein, and other protein cleavage events are also thought to play a role in neurodegeneration. Derivatization is often used to chemically modify specific amino acids in proteins prior to or after digestion, for example to prevent cleavage by preparative proteolytic enzymes (e.g. trypsin) used prior to MS and/or to alter the properties of residues on these peptides such that they can be subjected to affinity purification or enrichment, and also to improve the physical properties of the derivatized peptide for detection by MS. In most MS workflows, cysteine residues are routinely reduced and alkylated with iodoacetamide or N-ethylmaleimide to improve the identification rate of peptides including this residue. However, derivatization of other residues such as C-terminal ends and carboxyl groups on acidic side chains of Asp and Glu [48, 49] or primary amines on N-terminal ends of proteins and Lys side chains [50] has proven useful for the affinity enrichment of such *in vivo* N-terminal and C-terminal derived peptides. One approach has been termed terminal amine isotopic labelling of substrates (TAILS) [50]. Intact proteins are derivatized prior to preparative proteolytic digestion by trypsin, followed by a second, differential derivatization of enzyme-generated neo-N- and C-termini with a tag that can be affinity depleted by a binding matrix. The flowthrough peptides are then analysed by MS and represent peptide identifications of *in vivo* protein N- or C-termini, which can prove definitive for the identification of proteolytic cleavage occurring prior to isolation of the intact proteins. These approaches have opened up exciting possibilities both for identification of N-terminal PTMs such as cyclization and N- and C-terminal truncation by endogenous peptidases, respectively contributing to knowledge of the secretome [50] and degradome [51, 52].

#### 2.3.2 Histone derivatization

Epigenetic mechanisms have been implicated in neurodegenerative diseases including HD and AD. Histones store epigenetic information in the context of chromatin and for this reason, are functionally and dynamically decorated by a broad array of PTMs encompassing many, if not most, of the full array of enzymatically-derived PTMs [53]. Another application of derivatization is the modification of pre-enriched histones, which can be readily purified by affinity columns or even via extraction in strong acids. Histones are propionylated on N-termini and primary amines (Lys side chains) by propionic anhydride [54, 55]. This protects lysine residues from digestion by trypsin, and improves the mass/charge ratio of the longer peptides which would otherwise have too many positive charges if not for the chemical modification of positive charge-carrying primary amines into charge-free amides. Because longer peptides are generated by this approach, analysis that incorporates MS downstream is sometimes referred to as middle-down proteomics. Analysis of histones in bulk from whole cell chromatin averages the quantitative contribution of different PTM-specific peptides found on different DNA locus-specific regions of chromatin, thus the future of MS-based identification of histone PTMs that implicate those PTMs in regulation of specific DNA regions or genes will rely on innovative approaches that are still under development as they would apply to human samples [56, 57] with limited amounts of starting material [58].

#### 2.3.3 Examples of other PTM-oriented approaches benefiting from derivatization

Iodoacetamide, which alkylates cysteines at room temperature, also di-alkylates lysine side chains at elevated temperature, and this modification mimics the mass and charge characteristics of the tryptic remnant of ubiquitin attachment to lysine, diglycine [32], as mentioned above. This property has been successfully applied to tag all MS-accessible lysine residues of a purified protein in vitro to generate standard peptide MS/MS fingerprints of the protein to validate *in vivo*-generated ubiquitination attachment site peptides [59]. This is just one example of how derivatization can be used in innovative approaches for MS identification and validation of PTMs. In the future, it is foreseeable that derivatization and affinity purification may be increasingly combined to achieve purification and identification of novel PTMs. One example follows.

Secretion of proteins from tissue, such as from the brain into CSF, is a major source of biomarkers in living subjects. Analysis by MS of proteins secreted from cells, tissue, or organisms under certain physiological or pathological conditions is referred to as secretomics. The secretome typically incorporates PTMs such as signal peptide cleavage, among others, and constitutes an important class of proteins that control and regulate multiple physiological processes such as endocrine, paracrine, and autocrine signaling. This makes it a clinically relevant source for biomarkers and therapeutic targets. Although promising, most secretome studies were carried out using *in vitro* tissue rather than *in vivo*[60]. *In vitro*, both contamination by nonstandard secreted proteins and release of intracellular proteins contribute to the proteome detected [61]. However, derivatization shows promise in overcoming these problems. As mentioned above, N-terminal peptide derivatization for identification has been applied to the secretome, and there is also a fascinating development recently in a method called secretome protein enrichment with click sugars (SPECS), which identified veritable *in vivo* beta secretase substrates in brain [62], using exactly the combination of derivatization and affinity purification mentioned above.

### 2.4 Identification by orthogonal parallel approaches, e.g. oxyblot/MS

Oxidative stress resulting in oxidative damage of proteins is a common event in neurodegeneration. A number of studies [63–73] utilize parallel detection by, for example, two-dimensional polyacrylamide (2D-PAGE) oxyblot analysis, in which carbonyl moieties (aldehydes and especially ketones, both of which normally are not present on proteins) are reacted with dinitrophenylhydrazine to form dinitrophenylhydrazone (DNP) adducts. Then, following two-dimensional electrophoresis and transfer to membrane, a DNP-recognizing antibody is capable of detecting oxidation event(s) on one or more protein spots, where each spot is thought to represent a single, unique protein species. Then, in parallel analysis, these studies proceed to identify the same gel spots from a duplicate stained gel using MS [9]. Other versions of this parallel detection approach have been applied to the identification of, protein adducts occurring downstream of lipid peroxidation, where Michael adducts of alpha, beta-alkenal products of free radical mediated lipid peroxidation can attack proteins [69], and other modifications, such as advanced glycation end product protein conjugates. Since these modifications of proteins occur stochastically, and often on any of the 20 amino acid residues—as is the case for carbonylation—with limited site specificity as opposed to other PTMs which are imparted with enzymatic specificity, the abundance of any one chemically pure modification for detection by MS is limited, and thus a “guilt by association” approach may be the best available method for detection of these PTMs. A parallel immunoblotting/MS approach has also been used to examine proteins with high or enhanced 3-nitrotyrosine in AD brain as early as 2003, whereby this modification downstream of reactive nitrogen species (RNS) was implicated in potential dysregulation of neuronal metabolism and neuropeptide signalling [74]; a similar antibody against 3-nitrotyrosine can also affinity purify proteins prior to MS analysis [53]. There is an exception to the above where a specific non-enzymatic PTM can be directly detected by MS: methionine oxidation to methionine sulfoxide occurs within the range of physiological conditions, but oxidation and/or reductive loss of this PTM may also occur during protein and peptide processing steps, making it challenging to attribute to conditions *in vivo* with certainty. Nonetheless, it has been postulated that the microenvironment of cells affected by neurodegeneration is susceptible to attack of proteins and other molecules by reactive oxygen species (ROS) or RNS, downstream of not only contextually normal metabolic processes but also and especially following exposure to environmental toxicity which may precede loss of beneficial (or gain of toxic) function on the part of the modified protein [75].

### 2.5 Peptide affinity purification

The principles of affinity purification have also been successfully applied to the enrichment of specific PTM-decorated peptides following preparative proteolysis, thereby separating peptides which harbor the site of PTMs from others, even those derived from the same protein but lacking the PTM. Here we focus on one physicochemical approach at enriching phosphopeptides and a more general antibody-based approach at PTM-specific peptide pre-enrichment prior to MS. Such enrichment approaches are also envisioned to be useful protocols that could be coupled to other downstream detection, identification and/or quantitation methods.

#### 2.5.1 Phosphopeptide enrichment

Kinase dysregulation resulting in protein phosphorylation is widely implicated in neurodegenerative mechanisms, where extensive Tau phosphorylation preceding polymerization downstream of a host of kinases is just one example. Phosphorylated Ser, Thr, and Tyr residues with a high negative charge density have a particular affinity for transition metal cation chromatographic resins, such as germanium, titanium, niobium, hafnium, zirconium, and iron [76, 77]. Calcium chloride precipitation of phosphopeptides has also been explored with some success [78]. In a recent study, we employed immobilized metal affinity chromatography (IMAC) using ferric chloride bound to nitrilotriacetic acid (NTA) beads to achieve 80% purity of phosphopeptides extracted and digested from human post-mortem brain (Dammer et al., *Proteomics, in press*). While this study identified over 5,000 phosphopeptides, other studies using phosphopeptide enrichment prior to MS have identified well over 10,000 unique phosphosites across the proteome of a single cell line or tissue [79–81]. Other studies have focused on a smaller number of phosphosites in subcellular organelles, such as mitochondrial, phosphoproteomes in different tissues [82, 83], or the kinome [84, 85], which profiles functionally relevant phosphosites, usually on kinases themselves. The kinase activity assay for kinome profiling (KAYAK) approach has been recently optimized, even obviating the need for IMAC enrichment prior to detection and quantification of target kinome phosphopeptides [84].

#### 2.5.2 Antibody-based PTM- and/or motif-specific peptide affinity purification

To monitor how multiple critical signaling pathways are altered under specific conditions, an immunoaffinity-based enrichment step coupled to LC-MS/MS detection and quantitation called PTMScan has been developed and made commercially available as a trademarked product and service by Cell Signaling Technologies. Thousands of PTM-containing peptides from specific proteins participating in signaling pathways or specific protein types from cell lines, tissues, or xenografts can be identified utilizing the specificity of PTM- and/or motif-specific antibodies. The method is also compatible with both SILAC and label-free quantification. Thus far, target motifs include a series of PTMs specific to modifications occurring in signaling pathways, such as serine/threonine kinase specific motifs, tyrosine kinase substrate motifs, the Akt/PI3K pathway, lysine acetyltransferase substrates, and ubiquitination [86, 87]. The PTMScan concept represents a powerful quantitative method that is exceedingly adept at identifying, for example, ubiquitin diglycine attachment site remnant peptides via a specific antibody to enrich and purify tens of thousands of unique modified peptides that result from trypsin digestion of ubiquitinated proteins [33, 34].

## 3. A proteomic perspective of neurodegeneration: advances highlight PTMs in disease-defining aggregates, implicating quality control (QC) signalling and degradation deficits or gain of function

Proteomic advances have elucidated the molecular identity and specificity of different neurodegenerative diseases, often by identifying the proteins which are misfolded and thought to be—and often, later shown to be—toxic to cells in the CNS either through gain or loss of function due to the misfolding propensity of protein. PTMs regulate QC of misfolded proteins, and both their aggregation and clearance propensity [88]. Misfolding and aggregation are not, however, synonymous [89]. The latter process can be shown in certain cases to decrease toxicity of monomeric and oligomeric misfolded proteins, and is often an actively regulated process which relies on PTMs and related signalling prior to the deposition of PTMs, where aggregation can precede targeted degradation [90]. It is aggregates and their PTMs which are most easily detected in neurodegenerative disease tissue, thus they are considered the defining features of molecular pathology in neurodegenerative CNS [90]. In some cases, a PTM is obligate for the formation of a specific protein aggregate, for example, beta- and gamma- secretase cleavage of amyloid precursor protein is widely thought to be a necessary prerequisite to the formation of extracellular beta-amyloid-containing plaques that are a hallmark of AD, but which do not contain intact amyloid precursor protein, and rather only 40–42 residues of post-translationally cleaved sequence [91].

### 3.1 Stress-related PTMs, protein misfolding and the detergent resistant proteome

The cause(s) of protein misfolding [89] and its effects are both considered as forms of stress to cells, often with particular effects in specialized cells of the CNS. These forms of stress can range from mutation of the gene for the misfolding-prone (or aggregate prone) protein, aberrant signalling (often resulting in protein PTMs), metabolite or metabolism by-product accumulation or deficiency, or insufficient capacity to refold or clear toxic misfolded proteins through degradation pathways [92, 93]. These stresses often overlap or feed forward, enhancing each other and synergizing, accelerating decline in the function or viability of cells particularly in the complex milieu of CNS tissue, where many cells have extensive processes—e.g. dendrites and axons of neurons—isolated away from the cell body, which require local synthesis and transport of essential factors to remain fully functional, and disease associated proteins often have roles in these transport processes [90]. Compounded stress results in higher aggregation propensity for an affected protein and also often sequesters their usual, or novel, interaction partners. This aggregation-prone response to stress of certain proteins has been harnessed by some biological processes, for example polysome/messenger RNA (mRNA) stress granule formation, which is a response to oxidative or metabolic stress that temporarily arrests housekeeping gene translation in favor of stress responsive transcript translation; it happens that mRNA stress granule components contribute to detergent insoluble protein deposits in a number of neurodegenerative diseases [94]. Aggregation propensity of a protein is related to the ability of a structure to self-organize, such as mRNA stress granules or paired helical filaments including abundant tau protein in tauopathies, but such structure formation may involve a number of other protein and non-protein factors that have roles in templating and supporting extensive aggregation, often because they harbor intrinsically disordered regions with exceptional, if not also conditional, aggregation propensity [95] that can be modulated by PTMs such as phosphorylation [96]. The initial and complete identifications of the makeup of aggregates in disease have been major hurdles to progress in understanding how cells deal with stress, where one measurable outcome of that stress is the misfolding and subsequent aggregation of select proteins. But while protein aggregation is a signature of cellular stress responses, following the detection and identification of proteins in aggregates, an additional hurdle for researchers is to further elucidate the often unclear function(s) of the aggregate prone proteins and often, their associated intrinsically disordered domains which have thus been implicated in stress responses. Other factors and proteins which do not aggregate are also not excluded from important roles in stress response. However, aggregate prone proteins are particularly relevant to pathogenesis because in the case of proteinopathies which include AD, PD, tauopathies, and diseases with RNA binding protein inclusions, the aggregates are themselves pathogenic, in the sense they spread from an initial region of high vulnerability and incidence to brain regions progressively affected later during disease progression [97].

Historic milestones in publications implicating PTMs in disease-specific protein aggregation abound. Phosphorylated, ubiquitinated, acetylated, or oxidized species of pathogenic inclusions can partition differently into a detergent insoluble (hereafter “insoluble”) biochemical fraction, often with elevated levels in this insoluble fraction. Phosphorylation of insoluble alpha synuclein found in Lewy bodies of PD patients at a distinct residue (Ser129) increases *in vitro* aggregate formation four-fold [98], and can be deposited by a kinase genetically linked to PD [99]. Paired helical filaments that make up neurofibrillary tangles of affected neurons in AD are hyperphosphorylated and ubiquitinated [100], and recently have been associated within soluble U1 spliceosome components [23, 101, 102], in addition to classical hyperphosphorylated tau with this phosphorylation reducing Tau microtubule crosslinking character [103]. Lysine acetylation of Tau also occurs in the context of different peptide motifs within, to either prevent [104], or enhance [105, 106] accumulation of insoluble Tau fibrils. The mRNA stress granule protein TDP-43 is also hyperphosphorylated, cleaved, ubiquitinated, and/or oxidized, all preferentially in the insoluble fraction of neurodegenerative disease tissue and cellular models of TDP-43 inclusion or aggregate formation [59, 107–109]. Amyloid precursor protein is itself phosphorylated at T654, which is critical for subsequent cleavage into amyloid beta in a cellular neuronal AD model [110]; other PTMs occur on amyloid cleavage products, as well, including oxidation, phosphorylation, nitration, racemization, isomerization, pyroglutamylation, and glycosylation–involved in different physiological and pathological amyloid properties that may regulate disease progression [111]. An unconventionally translated hexanucleotide repeat expansion in C9ORF72 and mutant prion-like hnRNPA2 in familial forms of ALS syndromes also each contribute aggregate prone peptides to the insoluble fraction of select brain regions such as cerebellum and hippocampus, where the inclusions are positive for ubiquitin and ubiquitin binding proteins [112, 113]. The list in this paragraph is only representative, and by no means exhaustive, of aggregate prone protein PTM participation in insoluble aggregates found to be specific to different neurodegenerative diseases.

### 3.2 Ubiquitination, key to most misfolded protein and organelle QC mechanisms

Ubiquitin is an 8 kDa protein with a very stable fold resistant to misfolding, which makes this and 17 other ubiquitin-like protein folds which attach to substrates [114]. Ideal tags that can be used to sort and regulate misfolded or misfolding-prone proteins. Ubiquitin, SUMO, and interferon stimulated gene ISG15 are upregulated to deal with cellular or systemic stress such as reactive oxygen-induced misfolding of proteins, ER-associated stress, or an increase in protein misfolding propensity brought on during the heat shock response, whereas others may have divergent, more specialized, roles for example in signalling or enforcing enzymatic activity as does NEDD8 neddylation of cell cycle-stage specific cullin-ubiquitin ligase complexes, thereby activating substrate specific ubiquitin ligase activities during cell division. PTMs play a wide-ranging set of roles in QC, reviewed recently by Wang, Pattison and Su [115]. A vast array of ~600 ubiquitin ligases can deposit monoubiquitin or polyubiquitin chains on lysine residues or N-termini of tens of thousands of substrates across the human proteome, and ~98 deubiquitinases (endoproteases or isopeptidases) reverse this process, edit polyubiquitin signals, and/or recycle ubiquitin from its substrates [114] with dynamics that regulate detection and marking of misfolded protein, active refolding, sequestration of stubbornly misfolded proteins, and ultimately also as a fait accompli mark on aggregates, misfolded proteins, and dysfunctional whole organelles like mitochondria and peroxisomes, signalling their respective modes of destruction, thus limiting toxicity of misfolded protein and maintaining proteome and cell integrity via a cycle of protein QC interactions. Deubiquitinases are essential for initial ubiquitin release from multiple translation products of genes which encode tandem ubiquitin fusions, implicating the central importance of these along with ubiquitin in maintaining cellular QC capacity essential for survival. There is an essential hydrophobic patch on ubiquitin centered around Ile44, which mediates (poly)ubiquitinated substrate recognition and binding ubiquitin recognizing proteins, such as those containing UBA domains [116], which are often found in proteins that also harbor ubiquitin-like folds—thought to self-modulate interactions with ubiquitinated substrates. QC processes remain incompletely characterised due to the many niches which proteins occupy in a cell during their various life cycles and a division of labor between QC [117] and signal-dependent protein degradation—which targets correctly folded proteins at the time their function or activity must end such as during progression through the cell cycle. Despite this, much progress has been made by studying conserved QC mechanisms in eukaryotic systems with a very well characterized proteome, such as yeast—though often directly implicating roles for ubiquitin mediated protein QC and ubiquitin recycling in neurodegeneration. Invariably, the life cycle of a protein begins with co-translational quality control as any nascent polypeptide forms and exits the ribosome [118–120], and ends with terminal sequestration in insoluble aggregates or—more often in cells able to survive—degradation and recycling of components at the lysosome or proteasome—both highly conserved eukaryotic degradation systems. Ubiquitinated substrate degradation by the 26S proteasome relies on K48-linked polyubiquitin containing at least 4 ubiquitin moieties [121], but can utilize other unconventionally assembled polyubiquitin chains [32]. Proteasome insufficiency in neurons is enough to cause neurodegeneration and results in characteristic inclusions such as ALS-like Bunina bodies, Lewy body-like inclusions reminiscent of PD, and can exacerbate HD pathology [122–124]. Ubiquitin recycling which regulates ubiquitinated substrate degradation rate via a proteasome-associated deubiquitinase, Usp14, is also essential for maintenance of healthy synapses [125], where synaptic damage has come to define AD and PD pathogenesis [126], often downstream of glutamatergic dysregulation. For example, excitatory amino acid transporter has been shown to accumulate in biochemically insoluble lesions in AD, correlating with cognitive decline [127]. The enigmatic but abundant ubiquitin carboxy-terminal hydrolase also with potential ubiquitin ligase activity known as UCH-L1 was shown to be prone to oxidative modification(s) in AD brain in pioneering work by Castegna et al. [128]. This change may upset the activit(ies) of UCH-L1 such that the net balance of free-to-conjugated ubiquitin, or of edited ubiquitin linkages, is disrupted in AD brain [30].

Lysosomal targeting of cellular cargo into double-membrane enclosed autophagosomes uses a protein with an ubiquitin-like fold, light chain 3 (LC3) that is transferred to the autophagosome membrane via acyl (phosphatidylethanolamine) conjugation in a process that is reminiscent to ubiquitin activation (E1), conjugation (E2), and ligase (E3) activities. Following this, LC3 decorated preautophagosome membranes and monoubiquitin or ubiquitin chains on autophagosomal cargo are bridged by proteins like NBR1 or p62/sequestosome [129, 130], and then the mature, membrane enclosed, autophagosomeis fused with acidified vacuoles which ultimately mature into a cellular lysosome. Ubiquitinated substrate receptors like p62 can compete with or possibly cooperate with the proteasome for ubiquitinated substrate binding and degradation [131], but ubiquitin targeted autophagy appears to preferentially use K63-linked polyubiquitin chains, in addition to monoubiquitination, and K63 ubiquitination has been implicated in neurodegeneration [30, 132]. Autophagosomes which do not successfully degrade in lysosomes recapitulate granulovacuolar degeneration phenotypes seen in neurodegenerative disorders, particularly AD [133]. NBR1- and p62-positive pathology are also hallmarks of various insoluble protein deposits in neurodegeneration [134, 135]. Monoubiquitin attachment to select proteins displayed on mitochondria or peroxisomes also can be a sufficient signal for the autophagosomal targeting and degradation of these organelles [136]. The ubiquitination site specificity and topology of Parkin-dependent target modification in response to mitochondrial depolarization in PD has been examined using peptide affinity capture coupled to MS, revealing extensive conservation of Parkin-dependent ubiquitination sites on cytoplasmic domains in mitochondrial outer membrane proteins [137], consistent with a role for these residues in enabling mitophagy following a mitochondrial permeability transition [138]. Of note, direct substrate ubiquitination is excluded from some targeted degradation pathways involved in clearing misfolded proteins such as chaperone mediated autophagy, but there are a limited number of such substrates intrinsically defined by primary sequence motifs that regulate protein life span in conjunction with misfolding that may expose these often buried motifs [115]. Like the number of enzymes involved in ubiquitination, the cellular QC field is vast, and we refer the reader to reviews highlighted in this section for thorough coverage [92, 114, 115, 117, 139].

### 3.3 Signalling through receptors and kinase cascades, the secretome and roles of protein cleavage

We use the term signalling here in the context of pathways relevant to neurobiological function including, but not limited to: neuronal excitation- or activity-responsiveness, signalling between organs or within the CNS such as through endocrine or neuro-peptides, responsiveness to metabolite or nutrient availability, cellular growth, differentiation, extended cellular process formation, mRNA transport and mRNA translation to protein in such processes, glial responsiveness to changing tissue conditions in the CNS, inflammation, and last, broad programs of transcriptional regulation which can be a retrograde effector, if not also upstream of any of the above signal-dependent stimuli and responses. Rather than exhaustively reviewing the literature on this subject, we only touch on examples which have been put forward through the innovative use of proteomic approaches centered on PTMs or enzymes imparting PTMs that are likely to expand in their number of applications, use, and importance in coming years. For example, the first proteomic analysis of phosphorylated proteins in AD brain (hippocampus) was performed by Di Domenico et al. in 2011, finding that 17 proteins involved in neuronal energy metabolism and signalling pathways were significantly altered [140].

Neurotrophic signalling and activity-based stimulation of changes in the PTM status of downstream target proteins represent mediators of both neuronal outgrowth or axons and dendrites, and of memory consolidation. For example, following brain derived neurotrophic factor (BDNF) or Wnt stimulation of receptors on neurons, or as a quick response following neuronal depolarization, cyclic AMP-response element binding (CREB) protein is phosphorylated at Ser133 [141], promoting retrograde signalling to the nucleus via nuclear translocation and interaction with DNA response elements on downstream genes and with histone acetyltransferases like CREB binding protein CBP and other transcriptional coactivators, thereby promoting target transcription and local translation (confirmed by proteomics [142]) required for long term memory consolidation. O-GlcNAc modification of CREB Ser40 was discovered and found to be a central regulator in this process using enzymatic labelling of GlcNAc-terminated proteoglycan chains with a 2 kDa polyethylene glycol tag to enable detection of the PTM by Western blot, and confirmation was performed using electron transfer dissociation (ETD) MS, followed by numerous experiments elucidating an inhibitory role in neurite outgrowth, fear conditioning, and antagonism of coregulator binding to CREB [141]. An approach using azido-tagged sugar enzymatic modification of O-glycosylated peptides prepared for MS has also been described [143] and should gain in use by MS labs focused on this PTM in the future. O-GlcNAc also extensively modifies Tau [144] and this modification hinders aggregation [145]. Of note, O-GlcNAc modifications occur due to hyperglycemic conditions in diabetes, which is a major risk factor for AD, and this PTM crosstalks with various other PTMs, particularly phosphorylation [146]. SUMOylation has been shown to be activity dependent in the neuronal synaptosomal fraction, modifying numerous proteins and promoting glutamate receptor internalization and endosomal cycling downstream of long term potentiation [147], thus unbiased interrogation of SUMOylation substrates in synaptosomes by proteomic methods is warranted. Other modifications including acylation (e.g. palmitoylation of growth associated protein 43) play important but incompletely defined roles in learning and memory [148], awaiting developments in proteomic approaches that target these PTMs. Tubulin is a very abundant protein making transport possible and providing the cytoskeleton which co-forms with neuronal processes, and PTMs have been extensively characterized in neurons, localized to different functional domains in the cell [149]. Recently, a novel PTM, polyamination, was detected by MS on tubulin from axons, deposited by transglutaminase [150]. Thus, neurons and synapses harbor a wealth of PTMs that greatly expand the repertoire of protein functions beyond directly encoded transcript sequence in genes, and many of these PTMs are likely as yet undetected and uncharacterized, but we predict will be found to play important roles in neuronal function, especially learning and memory.

Secretion of protein and peptide factors that affect processes and downstream PTMs in signalling throughout the CNS is also a major area of intense experimentation, because CSF is an accessible source of proteinaceous sample for prognostic, diagnostic, and therapeutic biomarkers in neurodegeneration patients. Above, we mentioned the SPECS method, which was applied to identify 34 beta secretase BACE1-derived substrates from primary cells [62]. Automated MS interrogation of the secretome is also becoming a reality [151]. Secreted peptides derive from targeted proteolytic cleavage, thus, proteolysis is also a key regulatory PTM of interest in normal and pathological processes in the brain. Proteolysis plays a role in diverse cellular processes including programed cell death, immune function, and development. There are many crucial proteins and their cleaved fragments aggregate in neurodegeneration [152], however, the mechanism underlying the proteolytic processing of many proteins remains unclear. Therefore, unbiased methods employed toward an understanding of degradomics [52], for identifying protease substrates and tracking the extent of cleavage, are sorely needed for furthering our understanding of the pathogenesis of neurodegeneration. Recently, Herskowitz *et al.* analyzed post-mortem human brain using a forward and reverse (*in vitro*) MS-based approach to find that asparaginyl endopeptidease (AEP) directly cleaves TDP-43 at seven sites, two of which were found in FTLD brain tissue [153].

From the field of chemical proteomics, activity based probes have become powerful tools for characterizing distinct protease activity, targeting only the protease(s) that are active against a specific substrate [154]. This approach has been coupled to a MS readout [155], and focuses on identifying the source of specific cleavage events which may be unknown in a given cell or tissue context, rather than often well-established cleavage targets. Natural product small molecules, metabolites and drugs can also be engineered to generate protein adducts which are suitable for determining pharmacological protein targets using an activity based probe approach [156], which promises to open up mechanistic understanding of drug-protein interactions in neurodegeneration and other fields.

## 4. Future perspective

While a battery of proteomic approaches are available to quantitatively analyse PTMs on tens of thousands of targets, the functional alteration that PTMs engender requires a larger toolbox of experimental approaches which demand the combination of MS based approaches with more diverse methods, in order to go beyond simple PTM and protein identification and quantification. For example, despite the progress in methods in the last 10 years relating to histone PTMs highlighted in an above section, reports of histone modification differences in the context of native chromatin from different cell types of neurodegenerative disorders using unbiased MS approaches is hard to find. Methods which couple biophysical and biochemical separation of histones and/or nuclei [21] to histone PTM identification in the brain [157] need to be performed to provide insight into the biology of chromatin modifications that occur globally and/or in a locus-specific fashion at specific genes or chromosomal regions. In the future, we plan to carry out studies of epigenetic alterations in brains of individuals with AD, MCI, and FTLD. PTMs including oxidation, phosphorylation, ubiquitination and proteolysis all require further study in experiments that hold great promise for the elucidation of pathogenic mechanisms of neurodegeneration. Application of the technologies we have outlined are essential tools for the systematic, if not unbiased, study of neurodegenerative disorders, and promise to reveal multiple mechanisms of pathological protein aggregation and identify therapeutic targets in the future.

## Authors’ original submitted files for images

Below are the links to the authors’ original submitted files for images. Authors’ original file for figure 1

## Abbreviations

AD: Alzheimer’s disease
AEP: Asparaginyl endopeptidease
APP: Amyloid precursor protein
BDNF: Brain derived neurotrophic factor
cPILOT: Combined precursor isotopic labeling and isobaric tagging
CREB: Cyclic AMP-response element binding
CSF: Cerebrospinal fluid
DLB: Dementia with Lewy bodies
DNP: Dinitrophenylhydrazone
2D-PAGE: Two-dimensional polyacrylamide
ETD: Electron transfer dissociation
FACS: Fluorescence activated cell sorting
FTLD: Frontotemporal lobar dementia
HD: Huntington’s disease
IMAC: Immobilized metal affinity chromatography
LC3: Microtubule associated protein 1 light chain 3
RNS: Reactive nitrogen species
ROS: Reactive oxygen species
PTMs: Posttranslational modifications
PD: Parkinson’s disease
PNGase: Peptide N-glycanase
SUMO: Small ubiquitin-like modifier
SPECS: Secretome protein enrichment with click sugars
TAILS: Terminal amine isotopic labelling of substrates
TUBEs: Tandem ubiquitin binding entities
UBA: Ubiquitin binding associated (domain).
