# Supplementary figures and images for: Proteomics of protein post-translational modifications implicated in neurodegeneration
Source: Transl Neurodegener. 2014 Oct 30;3:23. doi: 10.1186/2047-9158-3-23 (PMC4323146; doi:10.1186/2047-9158-3-23)

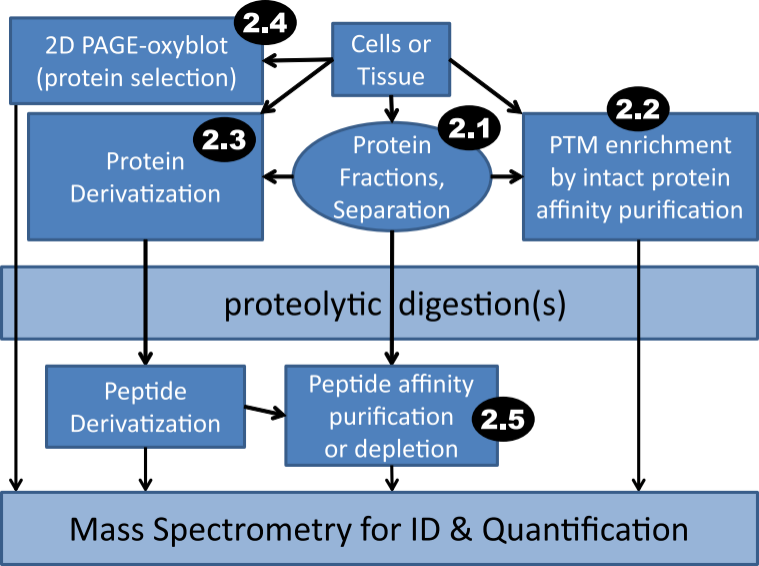

Supplement: Supplementary file 1 — Authors’ original file for figure 1 [file 40035_2014_70_MOESM1_ESM.pdf]
